# Intravenous iron isomaltoside 1000 administered by high single-dose infusions or standard medical care for the treatment of fatigue in women after postpartum haemorrhage: study protocol for a randomised controlled trial

**DOI:** 10.1186/1745-6215-16-5

**Published:** 2015-01-14

**Authors:** Charlotte Holm, Lars Lykke Thomsen, Astrid Norgaard, Jens Langhoff-Roos

**Affiliations:** Department of Obstetrics, Juliane Marie Centre, Copenhagen University Hospital, Rigshospitalet, Blegdamsvej 9, DK-2100 Kbh Ø Copenhagen, Denmark; Pharmacosmos A/S, Roervangsvej 30, DK-4300 Holbaek, Denmark; Section for Transfusion Medicine 2034, Capital Region Blood Bank, Copenhagen University Hospital, Rigshospitalet, Blegdamsvej 9, DK-2100 Kbh Ø Copenhagen, Denmark

**Keywords:** Postpartum haemorrhage, Iron deficiency, Intravenous iron, Postpartum fatigue

## Abstract

**Background:**

Postpartum haemorrhage can lead to iron deficiency with and without anaemia, the clinical consequences of which include physical fatigue. Although oral iron is the standard treatment, it is often associated with gastrointestinal side effects and poor compliance. To date, no published randomised controlled studies have compared the clinical efficacy and safety of standard medical care with intravenous administration of iron supplementation after postpartum haemorrhage.

The primary objective of this study is to compare the efficacy of an intravenous high single-dose of iron isomaltoside 1000 with standard medical care on physical fatigue in women with postpartum haemorrhage.

**Methods/Design:**

In a single centre, open-labelled, randomised trial, women with postpartum haemorrhage exceeding 700 mL will be allocated to either a single dose of 1,200 mg of iron isomaltoside 1000 or standard medical care. Healthy parturients with a singleton pregnancy will be included within 48 hours after delivery.

Participants will complete structured questionnaires that focus on several dimensions of fatigue and mental health (Multidimensional Fatigue Inventory, Edinburgh Postnatal Depression Scale and the Postpartum Questionnaire), at inclusion and at follow-up visits after three days, one week, three weeks, eight weeks, and 12 weeks postpartum. The primary endpoint is the aggregated change in physical fatigue score within 12 weeks postpartum, as measured by a subscale of the Multidimensional Fatigue Inventory. The primary objective will be considered to have been met if an intravenous high single dose of iron isomaltoside 1000 is shown to be superior to standard medical care in women after postpartum haemorrhage regarding physical fatigue.

For claiming superiority, we set the minimal clinically relevant difference between the mean scores at 1.8, and the assumed standard deviation at 4.2. Hence, 87 participants per treatment group are needed in order to demonstrate superiority; to provide an extra margin for missing data and dropouts, 200 women will be included.

**Discussion:**

The study will provide evidence on relevant clinical outcomes beyond biochemical parameters for intravenous iron isomaltoside 1000 compared to standard medical care in women after postpartum haemorrhage.

**Trial registration:**

This trial is registered with Clinicaltrials.gov (identifier: NCT01895218) on 26 June 2013.

**Electronic supplementary material:**

The online version of this article (doi:10.1186/1745-6215-16-5) contains supplementary material, which is available to authorized users.

## Background

### Postpartum iron deficiency and anaemia

Blood loss of up to 500 mL at delivery is considered to be a normal physiological mechanism that brings a woman’s haemoglobin value back to the pre-pregnancy level [1, 2]. Severe postpartum haemorrhage (PPH) is defined as blood loss above 1,000 mL within the first 24 hours after delivery [3]. Full body iron stores can replete blood loss up to a maximum of 1,000 mL [4], which means that many women with a blood loss between 500 and 1,000 mL will develop iron deficiency with or without anaemia. Therefore, in the present study of iron supplementation, we considered ≥700 mL blood loss to be the appropriate clinically significant criteria for inclusion.

Postpartum iron deficiency anaemia may lead to several clinical consequences, the most prominent of which is maternal physical fatigue [5]. It is currently unknown whether treatment can help this fatigue or whether biochemical markers, such as haemoglobin (Hb), ferritin and other biomarkers, can measure the condition and predict clinical treatment effects.

Breastfeeding is important for the health of the infant. Delayed onset of postpartum lactogenesis increases the risk of inadequate infant growth and early discontinuation of breastfeeding [6, 7]. A retrospective Canadian study found postpartum iron deficiency anaemia to be negatively associated with the duration of breastfeeding [8], but it is unknown whether postpartum iron deficiency anaemia is associated with delayed postpartum lactogenesis, or if the duration of breastfeeding can be improved with iron treatment after delivery.

The clinical consequences of postpartum iron deficiency without anaemia are not known in detail. However, iron deficiency without anaemia in women of childbearing age is associated with fatigue, impaired physical work performance, deficient cognitive functions and mood disturbances [9]. Therefore, it is possible that postpartum iron deficiency without anaemia contributes significantly to physical fatigue in the puerperium, and that iron treatment is beneficial for these women and their infants.

### Intravenous iron

The currently available intravenous iron preparations are generally considered equally efficacious but vary in terms of molecular size, kinetics, bioavailability and toxicology [10, 11]. The European Medicines Agency recently reviewed the safety of intravenous iron. It concluded that the benefits of intravenous iron are greater than their risks and was unable to establish a difference in the safety profiles of the products [12].

Isomaltoside 1000, the carbohydrate component in iron isomaltoside 1000 (Monofer®), has a mean molecular weight of 1,000 Da and consists predominantly of three to five glucose units. The carbohydrate isomaltoside is linear and unbranched with a low immunogenic potential. Iron isomaltoside 1000 has strongly bound iron within the iron isomaltoside formulation, which enables a controlled slow release of bioavailable iron to the iron-binding proteins with minimal risk of free iron toxicity [13].

To date, iron isomaltoside 1000 has been approved in Europe for the treatment of iron deficiency anaemia. The indications are when oral iron preparations are ineffective or cannot be used, or where there is a clinical need to deliver iron rapidly. Several studies of iron isomaltoside 1000 treatment of iron deficiency anaemia have been published, including the use of bolus injections and high single-dose infusions, without detected unexpected safety issues [14–17].

Iron isomaltoside 1000 was chosen for this study as it is approved for high single dosing of up to 20 mg/kg. The high single dose is preferable in the treatment of puerperal women, as they need fast correction of their iron deficit before leaving the hospital.

### Previous studies and current guidelines

International guidelines are available for the treatment of iron deficiency anaemia in pregnancy and the postpartum period [18–21]. These guidelines recommend oral iron supplementation in mild anaemia, intravenous iron in moderate anaemia and when oral iron supplementation is not tolerated, and allogeneic red blood cell (RBC) transfusion in severe and symptomatic cases. These guidelines do not deal with screening for or treatment of iron deficiency without anaemia.

Denmark has no published national guideline on the screening or treatment of postpartum iron deficiency with or without anaemia. In clinical practice, doctors usually recommend oral iron supplementation to parturients with PPH, and RBC transfusion to those with severe symptoms of anaemia. Since puerperal women with a Hb level of ≤6.5 g/dL (4.0 mmol/L) often have severe symptoms of anaemia, we will not include these parturients in the study.

No prospective randomised clinical studies have yet assessed the effect of intravenous iron supplementation in comparison to standard medical care in women after PPH with a clinical outcome as the primary endpoint. There have been 10 randomised controlled studies that have compared intravenous iron to oral iron supplementation in women with diagnosed postpartum iron deficiency anaemia [22–31]. The inclusion criteria of these studies were Hb concentration up to 10 days after delivery, and the primary study endpoints were Hb concentration, iron parameters and comparisons thereof. Generally, these studies have demonstrated improvement of Hb and iron parameters, favouring intravenous iron. With regard to safety, oral iron was associated with gastrointestinal side effects in up to 30% of the participants, with a compliance rate as low as 50%. These findings must be balanced against the very low risk of severe hypersensitivity reactions with intravenous iron [12].

Hb concentration and other biochemical parameters are influenced by the haemodynamic changes, and are therefore considered unreliable for diagnosing iron deficiency anaemia in the first week(s) after giving birth [32]. However, it is crucial that these women can benefit from treatment in the clinically important first days after delivery. In the present study, we have chosen to include women based on their clinically estimated blood loss after delivery, and we hypothesise that women with clinically significant PPH will have an increased risk of developing iron deficiency with or without anaemia.

We believe that iron treatment should be administered in order to improve clinical symptoms of iron deficiency with or without anaemia, not just to improve biochemical markers. This is in accordance with a systematic review, which concluded that there is a lack of high-quality studies with clinical outcomes [33]. Therefore, we have decided that the primary outcome in the present study will be a clinically significant improvement in the physical fatigue score between the treatment groups. We hypothesise that intravenous iron is superior to standard medical care.

### Objective

The primary objective of this study is to compare the efficacy of intravenous high single doses of iron isomaltoside 1000 to standard medical care on physical fatigue in women with PPH.

## Methods/Design

### Study design

The study will be a randomised, comparative, open-label single-centre study with a 1:1 allocation ratio. The trial is a company-sponsored interventional trial conducted according to International Conference on Harmonization-Good Clinical Practice (ICH-GCP) guidelines [34].

### Inclusion criteria

**Figure 1:**
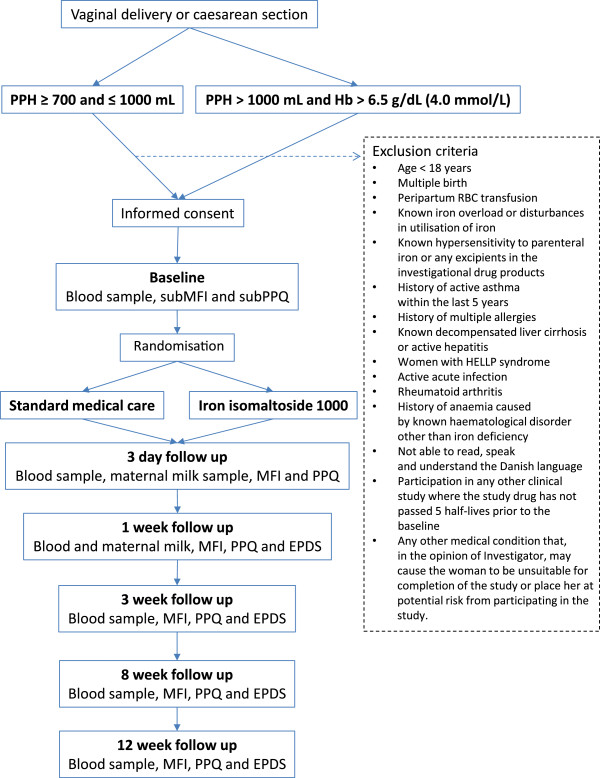
**Trial flow.** Women with postpartum haemorrhage ≥700 and ≤1,000 mL or PPH >1,000 mL and haemoglobin >6.5 g/dL (4.0 mmol/L) within 48 hours of delivery will be screened for inclusion and exclusion and asked for consent to participate. Baseline questionnaires are completed and blood samples are taken. The participants are randomised to either intravenous iron isomaltoside 1000 or standard medical care. Follow-up visits with completion of questionnaires, blood samples and maternal milk samples are performed at three days, and one, three, eight and 12 weeks after intervention. (EPDS: Edinburgh Postnatal Depression Scale; Hb: haemoglobin; HELLP: haemolysis, elevated liver enzymes, and low platelet count; MFI: Multidimensional Fatigue Inventory; PPH: postpartum haemorrhage; PPQ: Postpartum Questionnaire; RBC: red blood cells; subMFI: subscale of the Multidimensional Fatigue Inventory; subPPQ: subscale of the Postpartum Questionnaire).

Women, regardless of mode of delivery, will be eligible for inclusion in the study if they fulfil the following criteria: (1) they have signed the informed consent form, and (2) they have either (a) PPH ≥700 and ≤1,000 mL or (b) PPH >1,000 mL and Hb >6.5 g/dL (4.0 mmol/L) measured at >12 hours after delivery (Figure 1).

### Exclusion criteria

Exclusion criteria: age <18 years; multiple births; peripartum RBC transfusion; known iron overload or disturbances in the utilisation of iron (such as haemochromatosis or haemosiderosis); known hypersensitivity to parenteral iron or any excipients in the investigational drug products; a history of active asthma within the previous five years; a history of multiple allergies; known decompensated liver cirrhosis or active hepatitis; haemolysis, elevated liver enzymes, and low platelet count (HELLP) syndrome; active acute infection (clinical symptoms or fever above 38.5°C); rheumatoid arthritis; history of anaemia caused by a known haematological disorder other than iron deficiency; inability to read, speak and understand the Danish language; participation in any other clinical study where the study drug has not passed five half-lives prior to the baseline and any other medical condition that, in the opinion of the investigator, may cause the parturient to be unsuitable for completion of the study or place her at potential risk from participating in the study.

### Randomisation

Participants will be randomised using an interactive web response system (eClinical OS™, Merge Healthcare, Morrisville, NC, United States). The randomisation lists will be prepared centrally by the contract research organization BioStata ApS, Birkerød, Denmark using a validated computer program. The randomisation will be stratified by bleeding volume (PPH: 700 to 1,000 mL or PPH >1,000 mL). Within each bleeding category, there will be a 1:1 randomisation ratio to one of the two treatment groups.

### Intervention

The participants will be randomised to either standard medical care or iron isomaltoside 1000. Standard medical care (current treatment practice) at the Department of Obstetrics, Copenhagen University Hospital, Rigshospitalet is typically to recommend women with PPH to either continue oral iron supplementation, as during pregnancy (the Danish Health and Medicines Authority recommends 40 to 50 mg oral iron supplementation daily [35]), or to take 100 mg oral iron one or two times daily for a variable time period. The actual use of iron supplementation, including specific preparation, dose and duration of treatment, will be monitored at each follow-up visit.

Immediately after acute bleeding such as PPH, the Hb level is inaccurate for calculating individual dosing of iron supplementation. Hence, the iron isomaltoside 1000 fixed dosage in this study is based on the expected iron deficiency in women after PPH. Body iron stores are approximately 500 to 750 mg and the average iron loss through PPH is approximately 500 mg, as 500 mL blood loss equals 250 mg iron loss [4]. In previous studies of women with postpartum anaemia, oral iron treatment dosages were 130 to 200 mg per day over a period of 42 to 84 days, resulting in a total average dose of 11.088 mg [23–27]. The intestinal iron absorption varies considerably but is believed to be a maximum of 10% [36], resulting in a total expected absorption of 1,100 to 1,200 mg. Thus, the cumulated iron dosages in these studies are in accordance with the chosen treatment dose in this study.

In the group allocated to iron isomaltoside 1000, Pharmacosmos A/S, Holbaek, Denmark, the iron replacement dose is set to 1,200 mg. The dose is diluted in 100 mL 0.9% sodium chloride, Fresenius Kabi AG, Bad Homburg, Germany, and administered over a period of approximately 15 minutes. No test dose is applied. Participants with a pre-pregnancy weight <45 kg will receive a reduced single dose of 1,000 mg iron isomaltoside 1000, administered in the same fashion. In both treatment groups, participants may be given a ‘rescue’ allogeneic RBC transfusion if this procedure is clinically indicated.

### Setting, location and follow-up

The parturients will be screened for inclusion in the study over an 18-month period at the Department of Obstetrics, Copenhagen University Hospital, Rigshospitalet, a tertiary hospital that performs between 6,000 and 7,000 deliveries per year. Eligible parturients are informed about the study and must provide their written consent within 48 hours after delivery. The duration for each individual participant is approximately 12 weeks and involves five follow-up visits by a midwife at the participant’s home. The timeframes of the follow-up visits are three days (between two and four days), one week (between six and eight days), three weeks (between 19 and 23 days), eight weeks (between seven and nine weeks) and 12 weeks (between 11 and 13 weeks).

### Outcomes and safety measures

The primary outcome is the aggregated change in physical fatigue score within 12 weeks postpartum, as measured by a subscale of the Multidimensional Fatigue Inventory (MFI) [37]. Secondary efficacy outcomes include changes in the Hb concentration, *p-*ferritin, *p-*iron, *p-*transferrin, transferrin saturation, reticulocyte count, mean reticulocyte Hb content, other MFI and Postpartum Questionnaire (PPQ) fatigue items, symptoms of postpartum depression, time of postpartum lactogenesis, time of discontinuation of breastfeeding and transfusion of allogeneic RBCs.

The safety outcomes are discontinuation due to intolerance, frequency, nature and severity of adverse events, vital signs, and *p*-sodium, *p-*potassium, *p-*calcium, *p-*phosphate, *p-*urea, *p-*creatinine, *p-*albumin, *p*-bilirubin, *p*-aspartate aminotransferase and *p*-alanine aminotransferase levels. Other outcomes are maternal milk iron level, anaemia symptoms and gastrointestinal symptoms.

### Questionnaires

We will use the three following self-reported questionnaires: the fatigue measures MFI and PPQ and the depression measure Edinburgh Postnatal Depression Scale (EPDS) [38]. Of these, the MFI includes the scale of physical fatigue, which is the primary endpoint of this investigation.

The MFI has been found to have high feasibility, reliability and validity in chronically anaemic women [39] and in postpartum women [40]. The MFI covers the following dimensions: general fatigue, physical fatigue, reduced activity, reduced motivation and mental fatigue. The MFI consists of 20 statements for which the participant indicates, on a five-point scale, the extent to which the particular statement applies to her. The statements refer to aspects of fatigue experienced during the previous days. Higher scores indicate a higher degree of fatigue.

The PPQ is a novel and yet to be validated instrument that was developed for this study to measure fatigue in parturients [see Additional file 1 (in Danish)]. It consists of a visual analogue scale of how tired the participant is and five questions regarding fatigue. The first question concerns what the participant experiences as most troublesome, pain or fatigue. The second question concerns whether the fatigue is experienced most physically or mentally. For each of the first two questions the participant is asked to underline one of five possible responses. The last three questions explore how the fatigue influences breastfeeding, contact with the newborn and to what extend the participant asks for help with nappy changing. The participant is asked to indicate, on a four-point scale, the extent of the influence of the fatigue. Higher scores from the last three questions indicate a higher degree of fatigue.

The EPDS is a screening instrument that was developed to detect symptoms of depression in puerperal women. A number of studies have confirmed that the EPDS is both reliable and sensitive [41]. The EPDS consists of 10 questions. Participants are asked to underline one of four possible responses that comes closest to how she has been feeling during the previous seven days. The maximum score is 30 and a score of 10 or higher indicates possible depression.

### Monitoring and data collection

The data collection tool for this study is an electronic case report form. Data necessary for analyses and reporting will be entered into a validated and secured data system. Clinical data management is performed in accordance with applicable standards and data cleaning procedures.

A clinical research associate (CRA) from a contract research organisation will monitor the study for protocol compliance, verifying that safety procedures and the rights of participants are being protected. The CRA will also verify that the study is conducted in accordance with the currently approved protocol and any other study agreements, ICH-GCP guidelines and all applicable regulatory requirements. The CRA will also monitor and verify that data are authentic, accurate and complete.

### Number of participants

The following hypothesis will be tested: iron isomaltoside 1000 is superior to standard medical care in the treatment of iron deficiency in women with PPH with or without anaemia, measured primarily by a reduction in physical fatigue. The null hypothesis of no difference between the treatment groups will be tested against the alternative by constructing a two-sided 95% confidence interval of the difference in aggregated change in physical fatigue score from baseline to week 12 between the two treatment groups. The sample size calculations are based on the primary endpoint, aggregated change in physical fatigue score from baseline to week 12. A two-sided significance level of 5% is used and the power is set to 80%.

The use of the physical fatigue subscale of MFI allows a maximum change of 16 points. Based on clinical judgement and consensus between all authors, we wish to see a difference greater than 10% for claiming clinically relevant superiority. Hence, we set the minimal clinically relevant difference to 1.8. In a previous study, the standard deviation was found to be approximately 4.2 [40]. Therefore, 87 patients per treatment group are needed in order to demonstrate superiority. With a margin for missing data and a dropout rate of 13%, 200 women will be included.

### Statistical methods and data analysis

All tests are pre-specified in a statistical analysis plan. The primary analysis population will be the full analysis set, including all randomised participants who received the study drug and have at least one post-baseline physical fatigue score. The primary endpoint is the aggregated change in the physical fatigue score from baseline to week 12. This will be calculated as the area under the curve of the changes in the physical fatigue score from baseline to each visit until week 12, divided by the number of scheduled days (observation period). The area under the curve will be calculated using the linear trapezoidal method and scheduled days. The primary endpoint will be analysed using an analysis of variance model, with treatment and amount of PPH (700 to 1,000 mL or >1,000 mL) as factors, and baseline MFI physical fatigue score as the covariate. The estimated treatment differences (iron isomaltoside 1000 versus standard medical care) expressed as contrasts of the adjusted means will be presented with corresponding 95% confidence intervals and the *P* value. Iron isomaltoside 1000 will be considered superior to standard medical care if the lower limit of the 95% confidence interval is above zero. We will also perform a per protocol analysis excluding participants with major protocol deviations, such as rescue allogeneic RBC transfusion.

All statistical tests of the efficacy endpoints will be two-sided and performed on a 5% significance level. Estimated treatment differences and 95% confidence limits will be presented together with the corresponding *P* value. Continuous secondary endpoints will be analysed by a mixed model for repeated measurements, including visit and treatment-by-visit, and PPH (700 to 1,000 mL or >1,000 mL) as factors, the baseline value as the covariate, and the subject as the random effect. If no baseline value is measured for the endpoint in question, the value at the first measured time will be included as the covariate. An unstructured covariance structure will be used to model the within-subject errors and the estimation method will be a restricted maximum likelihood-based approach. The analysis will be based on the ‘missing at random’ assumption and performed using the data from each observed case. ‘Proportion’ endpoints will be analysed by logistic regression with treatment as the factor. Where relevant, the baseline value of the parameter in question will be included as the covariate. Time to postpartum lactogenesis and time to discontinuation of breastfeeding will be assessed and analysed using Kaplan-Meier curves and compared between treatments using a log-rank test. The laboratory data will be analysed as described for the continuous variables above. Adverse events will be summary tabulated using the latest version of the Medical Dictionary for Regulatory Activities body system and preferred term indicating the number and percentage of participants and the number of events. The number of participants who experience an adverse drug reaction, including suspected unexpected serious adverse reactions, will be compared between treatment groups.

### Consent and ethical considerations

Women planning to give birth at the Department of Obstetrics, Copenhagen University Hospital, Rigshospitalet have received written information about the study at one of their antenatal midwife consultations. When a parturient fulfils the inclusion criteria, she will be asked to participate in the study after receiving oral and written information from an investigator. In obtaining and documenting informed consent, the investigator will comply with any applicable regulatory requirements, and will adhere to ICH-GCP and the Declaration of Helsinki. The National Committee on Biomedical Research Ethics (approval number: H-4-2013-019) and the Danish Medicines Agency (approval number: EudraCT 2012-005782-12) have approved the study. The trial is registered at Clinicaltrials.gov (identifier: NCT01895218).

## Discussion

The aim of this study is to compare a new treatment option (iron isomaltoside 1000) to standard medical care. The choice of the latter active comparator, ‘the drug of first choice’, enables us to use the results of this study for assessing the potential additional benefit of iron isomaltoside 1000 in a clinical setting.

The standard medical care treatment group does not receive a general specified recommendation of oral iron supplementation. We considered that standard treatment, as defined by the individualised regimen chosen by the parturient and her midwife, was the proper comparator. A pregnant woman usually takes oral iron supplementation from early pregnancy and finds a preparation and a dose that is suitable for her. A general fixed regimen might cause gastrointestinal side effects in some individuals, and a possible insufficient effect in others. Therefore, the fixed regimen would be suboptimal as a comparator compared with the standard individualised regimen, which is current practice in the Capital Region of Denmark. The actual use of oral iron, including specific preparation, dose and duration of treatment, is registered at each follow-up visit.

The individualised regimen design implies that we were not able to blind the randomisation in this trial. Also, since iron tablets colour the stools, we assessed that a double-blinded design would be unfeasible and would not contribute additional scientific merit. Accordingly, most previously published randomised controlled trials with iron treatment of women after childbirth have not been double-blinded [23–27].

The strength of this trial is the fact that it will be conducted at a single centre. This means that the standard medical care is less diverse than it might otherwise be and we can ensure a full overview of all parturients with PPH ≥700 mL in the time period of inclusion, which will enable us to collect detailed information about those excluded and the reasons why not.

The risk of bias by selecting the subjective endpoint physical fatigue in an open-label study is an inherent weakness of the study. Fatigue is an important clinical endpoint that has consistently been shown to be affected by iron deficiency and iron deficiency anaemia. However, fatigue in the postpartum period is highly dependent on various attributes, including the mode and outcome of delivery, the duration of labour, medical comorbidities, the health of the infant and a host of social and environmental factors. Nevertheless, this design was previously used in a randomised controlled study in a similar population, where an open-label design and subjective primary endpoint were chosen for the most clinically relevant design [42].

The instrument used for measuring the primary endpoint is the physical fatigue subscale of the MFI. We selected this instrument due to its previous validation, including the findings of high feasibility and reliability in the postpartum population, specifically its use for measuring fatigue in parturients that experienced PPH. However, no validated questionnaires have been specifically developed for measuring postpartum fatigue. Although some available questionnaires for measuring fatigue have been validated for this population, they are all constructed to measure fatigue in other populations, often cancer or other chronic ill patients. Therefore, we developed a new questionnaire (PPQ) that is designed specifically to measure fatigue among puerperal women. A further objective of this study is to assess and validate its ability to measure fatigue in women after PPH.

## Trial status

The trial was initiated and the first parturient was included in May 2013. Inclusion and randomisation are ongoing and expected to be finished in August 2014.

## Electronic supplementary material

Additional file 1: **Postpartum Questionnaire.** (PDF 64 KB)

## Abbreviations

CRA: clinical research associate
EPDS: Edinburgh Postnatale Depression Scale
Hb: haemoglobin
HELLP: haemolysis, elevated liver enzymes, and low platelet count
ICH-GCP: International Conference on Harmonization – Good Clinical Practice
MFI: Multidimensional Fatigue Inventory
PPH: postpartum haemorrhage
PPQ: Postpartum questionnaire
RBC: red blood cells.
