# Supplementary material for: Intravenous iron isomaltoside 1000 administered by high single-dose infusions or standard medical care for the treatment of fatigue in women after postpartum haemorrhage: study protocol for a randomised controlled trial
Source: Trials. 2015 Jan 14;16:5. doi: 10.1186/1745-6215-16-5 (PMC4326197; doi:10.1186/1745-6215-16-5)
Supplement: Supplementary file 1 — Additional file 1: Postpartum Questionnaire. (PDF 64 KB) [file 13063_2014_2392_MOESM1_ESM.pdf]

POSTPARTUM QUESTIONNAIRE

1. Hvor træt er du nu? Angiv med en lodret streg på nedenstående linje hvor træt du føler dig.

   Ikke træt |________________________________| Meget træt

2. Hvad oplever du mest besværende, smerter eller træthed?

   Kun smerter – Mest smerter – Ligeligt – Mest træthed – Kun træthed

3. Hvordan mærker du trætheden mest – fysisk eller psykisk?

   Kun fysisk – Mest fysisk – Ligeligt – Mest psykisk – Kun psykisk

4. Føler du trætheden influerer på din amning?

   Meget – Noget – Lidt - Slet ikke

5. Føler du trætheden influerer på din kontakt til dit barn?

   Meget – Noget – Lidt - Slet ikke

6. Er du så træt at du må bede andre om at hjælpe med bleskift?

   Hver gang – Ofte - Af og til – Aldrig

Mange tak for din medvirken
